# Early psychological screening of intensive care unit survivors: a prospective cohort study

**DOI:** 10.1186/s13054-017-1813-z

**Published:** 2017-11-09

**Authors:** Anna Milton, Emily Brück, Anna Schandl, Matteo Bottai, Peter Sackey

**Affiliations:** 1 Department of Physiology and Pharmacology, Karolinska Institutet, Stockholm, Sweden; 2 Department of Perioperative Medicine and Intensive Care, Karolinska University Hospital, 171 76 Stockholm, Sweden; 3 Department of Molecular Medicine and Surgery, Karolinska Institutet, Stockholm, Sweden; 4 Department of Environmental Medicine, Karolinska Institutet, Stockholm, Sweden

**Keywords:** Critical care, Intensive care units, Stress disorders, Post-traumatic, Anxiety, Depression, Follow-up studies

## Abstract

**Background:**

A majority of patients survive their episode of critical illness but up to 30% of patients suffer from psychological problems such as post-traumatic stress, anxiety and depression in the year after intensive care unit (ICU) stay. A method to identify discharged patients at risk for adverse psychological outcome would be helpful in the triage for ICU follow-up and could enable early intervention. The aim of this study was to evaluate whether early screening with validated questionnaires after ICU discharge can identify patients at risk for symptoms of post-traumatic stress, anxiety and depression 3 months after ICU stay.

**Methods:**

We performed a prospective observational cohort study in the general ICU at the Karolinska University Hospital Solna, Stockholm, Sweden. All adult patients surviving ≥ 24 hours in the ICU in a 9-month period were eligible for inclusion. Patients with mental disability, serious auditory and visual disorder, aphasia or who were unable to understand Swedish were excluded. One hundred and thirty-two patients were included and visited by a follow-up nurse within 1 week after ICU discharge. The Hospital Anxiety and Depression Scale (HADS) and the Post-Traumatic Stress Symptoms Checklist-10 (PTSS-10) were administered. Three months after ICU discharge the patients received the same questionnaires by postal mail. We assessed the predictive values of the questionnaires using the area under the receiver operating characteristic curve (AUROC). For correlation calculations, we used Spearman’s rank correlation coefficient. Negative and positive predictive values for each questionnaire were calculated.

**Results:**

Eighty-two patients returned the follow-up questionnaires. We found correlation between early and late scores and reasonable predictive precision regarding 3-month outcomes, with an AUROC of 0.90 for PTSS-10 part B, 0.80 for the HADS anxiety subscale and 0.75 for the HADS depression subscale.

**Conclusions:**

Symptoms of post-traumatic stress, anxiety and depression assessed 1 week after ICU stay correlate with 3-month psychological outcome. The HADS and PTSS-10 may be useful aids to identify ICU survivors at high risk for clinically significant symptoms of post-traumatic stress, anxiety and depression 3 months post ICU stay.

## Background

The majority of intensive care unit (ICU) patients are admitted to the ICU due to unexpected and life-threatening illness or injury, impacting physical and psychological recovery. ICU survivors suffer considerable long-term complications from critical illness and ICU stay [1], including psychological problems [2]. In ICU follow-up studies, one in three ICU survivors has clinically significant symptoms of depression or post-traumatic stress disorder (PTSD) in the year after ICU stay [3, 4]. These problems usually persist for a long period of time and affect health-related quality of life [5, 6] but can potentially be managed if symptoms are recognised.

To facilitate the recovery of these patients, guidelines have been issued recommending ICUs to follow-up ICU survivors during the first year of critical illness. However, the evidence for such ICU follow-up is not consistent [7–9]. Yet, for vulnerable subgroups, follow-up has been found to improve patients’ psychological outcome [10, 11].

Ideally, follow-up would start during hospitalisation and also target specific anticipated problems in the individual patient [12]. Despite suggestions of such early follow-up, there are no formal methods to assess ICU survivors at an early stage after ICU stay in order to anticipate the long-term psychological outcome.

The aim of this study was to evaluate whether symptoms of post-traumatic stress, anxiety and depression assessed with two questionnaires in the week after ICU discharge can predict symptoms 3 months later.

## Methods

The study was approved by the Karolinska Institutet Regional Ethics Review Board in Stockholm, Sweden (approval number 2012/35-31/2).

### Study design

This prospective cohort study included survivors from one ICU in a tertiary-care hospital in Sweden. Patients’ psychological status was evaluated with an early assessment during the first week of ICU discharge, and at follow-up 3 months after discharge.

### Study population

All adult patients surviving to ICU discharge after more than 24 hours in the mixed surgical–medical general ICU at Karolinska University Hospital Solna during March 2012–March 2013, with a break during June and August, were eligible for inclusion. Patients were excluded if they were mentally disabled, had serious auditory or visual disorders, were unable to understand Swedish or suffered from aphasia.

### Data collection

An ICU follow-up nurse visited ICU survivors in the general ward within 1 week from discharge and gave them questionnaires assessing symptoms of PTSD, anxiety and depression. Patients filled out the questionnaires by pen and paper. In the few cases when patients were unable to write, the nurse read the questions and answer options out loud and filled out patients’ responses. Three months after ICU discharge the patients received the same questionnaires by postal mail. Non-responders received a reminder telephone call and a new set of questionnaires were sent 2 weeks after the first set. Data on patient characteristics were collected from the medical charts and patient data management system.

### Outcome

The Post-Traumatic Stress Symptoms Checklist-10 (PTSS-10) and the Hospital Anxiety and Depression Scale (HADS) questionnaires were used to evaluate symptoms of PTSD, anxiety and depression at both assessments. The PTSS-10 is a validated screening tool for the detection of PTSD-related symptoms among ICU survivors [13]. Part A consists of four questions concerning memories of traumatic events and feelings while in the ICU, such as nightmares, anxiety or panic, pain or trouble to breathe. Questions can be answered yes or no. Part B consists of 10 questions concerning ongoing stress symptoms. Each item is scored from 1 (never) to 7 (always) with a total score range from 10 to 70 points. A score above 34 in PTSS-10 part B indicates clinically significant post-traumatic stress symptoms and is associated with a diagnosis of PTSD [14].

The HADS is a questionnaire consisting of two subscales measuring patients’ symptoms of anxiety and depression. Each subscale consists of seven items scored from 0 to 3, resulting in a subscale score range from 0 to 21. A subscale score above 7 suggests clinically significant problems [15]. The questionnaire has been validated among general medical patients as well as critically ill patients [16, 17].

### Statistics

STATA version 12.1 (StataCorp LP, College Station, TX, USA) was used to analyse data. The alpha level was set to 5%. Median scores and interquartile ranges for the questionnaires were calculated. For calculation of monotonic correlations, we used Spearman’s rank correlation coefficient. We performed receiver operating characteristic (ROC) analyses to assess the predictive value of the early assessment with the area under the curve (AUROC). The ROC curves were also used to identify the optimal cut-off value for the early assessment with regard to sensitivity and specificity. Negative and positive predictive values for the questionnaires were calculated. The Mann–Whitney *U* test was used to compare continuous numerical variables of baseline characteristics and early questionnaire scores between responders and non-responders. For comparison of categorical variables, the chi-square test or Fisher’s exact test were used as appropriate.

## Results

**Fig. 1:**
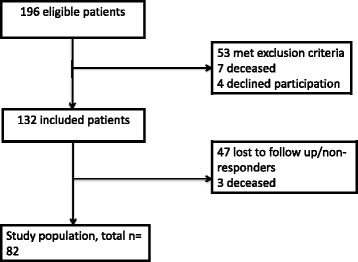
Flow chart of patient inclusion

A total of 132 patients were included, of whom 82 patients (62%) returned the follow-up questionnaires 3 months after discharge from the ICU (Fig. 1). Table 1 shows patients’ baseline characteristics. We found significant correlations between scores in the ward within 1 week after discharge and 3 months after discharge from the ICU in all three questionnaires (Table 2).

**Table 1:** Baseline demographics of included patients

Variable	Value
Female	55 (42)
Age (years)	62 (41–70)
Diagnosis category	
Medical	56 (42)
Surgical	57 (43)
Trauma	19 (14)
APACHE II score^a^	10 (7–14)
LOS (days)	3 (2–6)

Data presented as *n* (%) or median (interquartile range)

*APACHE* Acute Physiology and Chronic Health Evaluation, *LOS* length of stay

^a^Data missing on APACHE II score for nine patients

**Table 2:** Questionnaire scores and correlation between early assessment and follow-up

Questionnaire	Early scores among responders (*n* = 82)	Responders with scores above cut-off value at early assessment (%)	Three months’ scores	Patients with scores above cut-off value at follow-up (%)	Correlation (*p* value)
PTSS-10 B	20 (15–29)	15	17 (13–30)	13	0.60 (<0.001)
HADS anxiety	3 (1–7)	23	2 (1–5)	16	0.48 (<0.001)
HADS depression	4 (1–7)	23	4 (1–6)	21	0.56 (<0.001)

Scores presented as median (interquartile range) unless otherwise stated. Cut-off value for symptoms of PTSS-10 B is > 34 points. Cut-off value for symptoms of HADS Anxiety and HADS Depression is > 7 points. Correlation between early assessment and 3-month follow-up calculated with Spearman’s rank correlation coefficient

*PTSS-10 B* Post-Traumatic Stress Symptoms Checklist-10 part B, *HADS* Hospital Anxiety and Depression Scale

### Predictive accuracy of post-traumatic stress symptoms

**Fig. 2:**
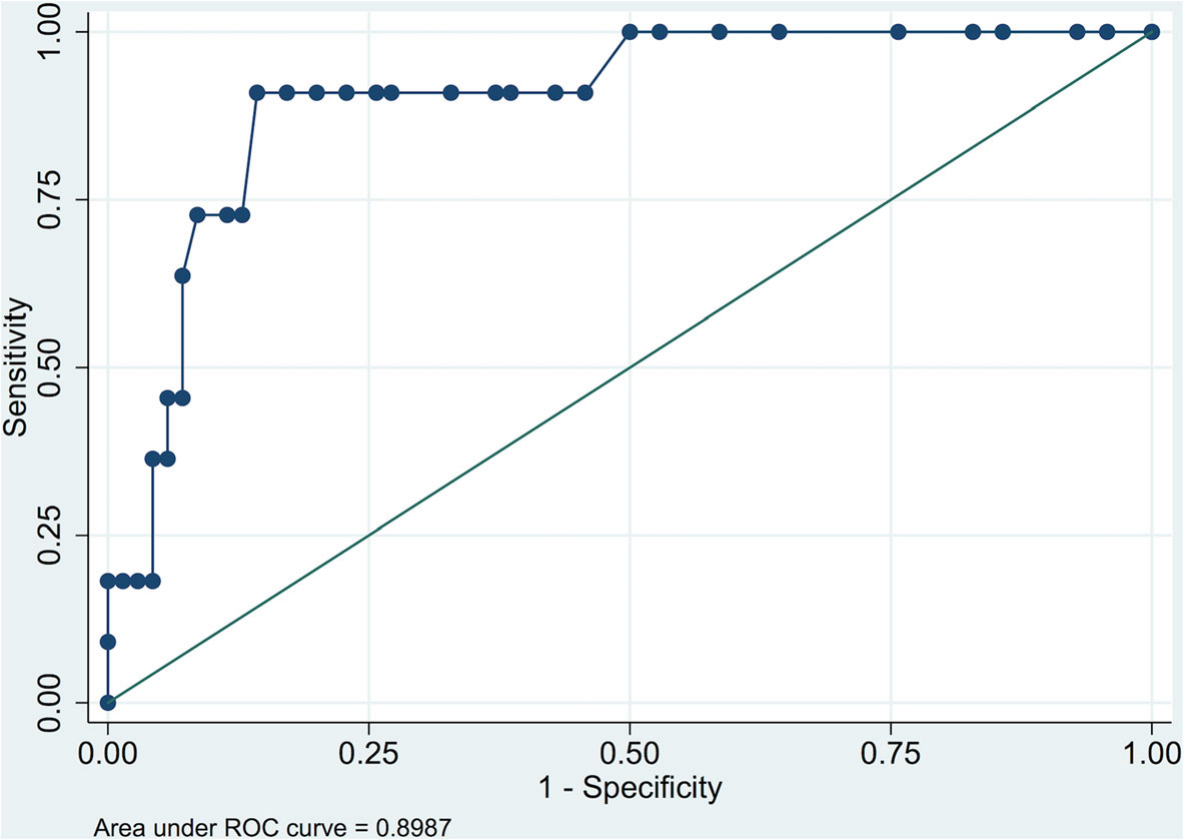
Area under the receiver operating characteristic curve for early assessment and 3-month outcome for PTSS-10 part B. *ROC* receiver operating characteristic

At 3 months, 11 patients (13%) had PTSS-10 part B scores > 34, implying clinically significant symptoms of post-traumatic stress. At this cut-off value for late symptoms of post-traumatic stress, the AUROC was 0.90 (Fig. 2). With a ward screening cut-off value > 29 points and caseness at 3 months defined as > 34 points, sensitivity was 91% and specificity was 86%. The positive predictive value (PPV) was 50% and the negative predictive value (NPV) was 98%.

### Predictive accuracy of anxiety symptoms

**Fig. 3:**
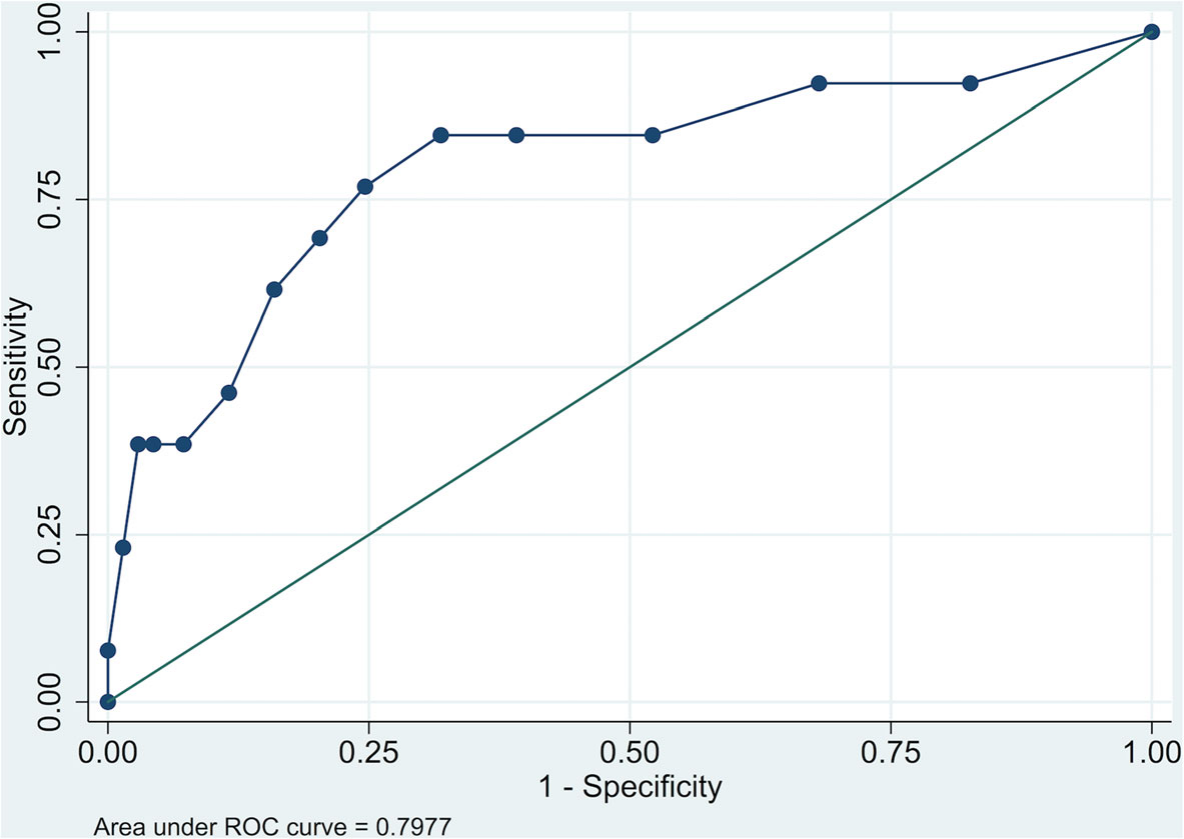
Area under the receiver operating characteristic curve for early assessment and 3-month outcome for the HADS anxiety subscale. *ROC* receiver operating characteristic

Thirteen patients (16%) had a HADS anxiety subscale score of > 7, implying clinically significant symptoms of anxiety 3 months after discharge from the ICU. This cut-off value for late anxiety symptoms generated an AUROC of 0.80 (Fig. 3). An early screening cut-off value > 5 points and 3-month caseness defined as > 7 points yielded a sensitivity of 77%, with a specificity of 75%. The PPV was 37% and the NPV was 95%.

### Predictive accuracy of depressive symptoms

**Fig. 4:**
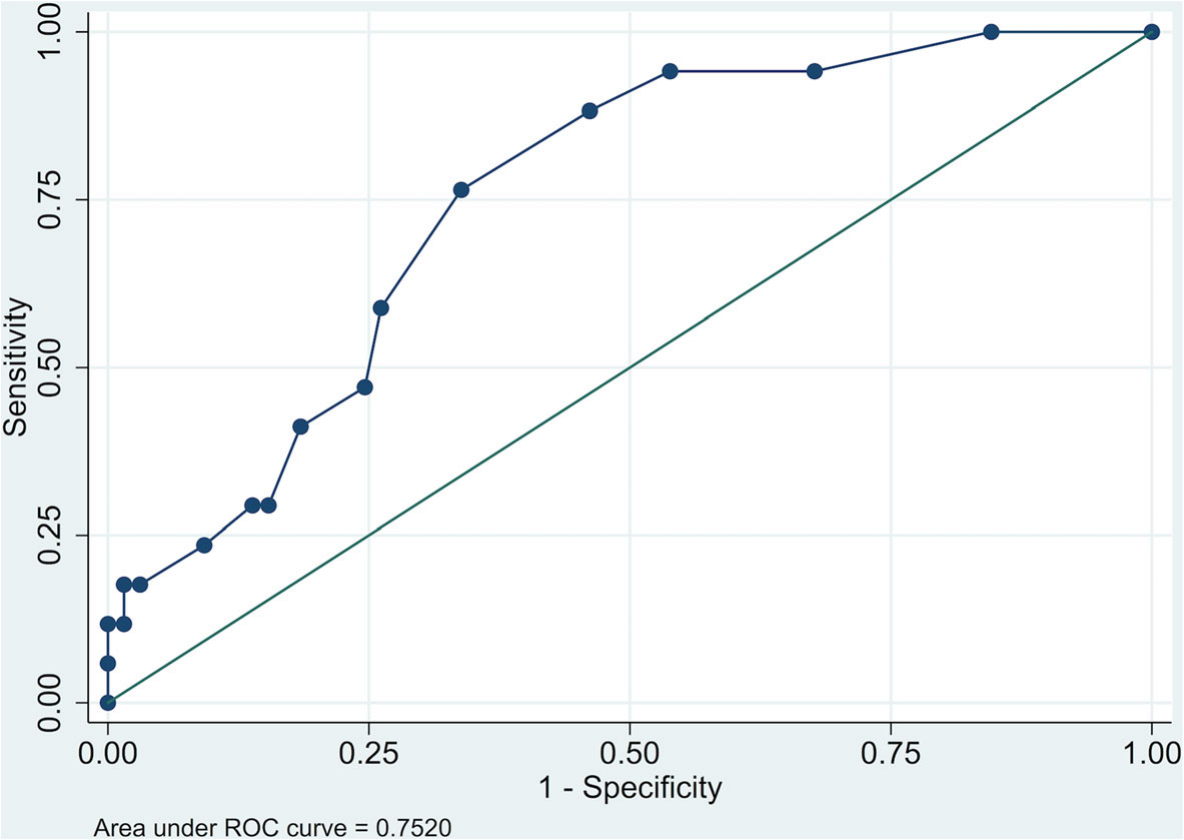
Area under the receiver operating characteristic curve for early assessment and 3-month outcome for the HADS depression subscale. *ROC* receiver operating characteristic

Seventeen patients (21%) had scores implying clinically significant symptoms of depression, defined as HADS depression subscale score > 7, 3 months after ICU discharge. The AUROC for late depressive symptoms was 0.75 (Fig. 4). An early screening cut-off value > 4 points and 3-month caseness defined as > 7 points yielded a sensitivity of 76% and a specificity of 66%. The PPV was 37% and the NPV was 91%.

### Association between early traumatic memories and late symptoms of post-traumatic stress

We performed a post-hoc analysis regarding the association between traumatic memories at the early assessment (PTSS-10 part A) and 3-month PTSS-10 part B scores. Fifty-one per cent of responders reported zero or one traumatic memory and 48% reported two to four traumatic memories. Twenty-eight per cent of patients reporting two or more traumatic memories scored above the predefined cut-off value in the 3-month PTSS-10 part B, while no patients reporting zero or one traumatic memories scored above the cut-off value (*p* < 0.001). The median score among patients with two to four traumatic events was 23 (IQR 16–35), while the median score among patients with zero or one traumatic events was 16 (IQR 12–23). The difference in median score between groups was also statistically significant (*p* < 0.001).

### Non-responders

A total of 50 patients (38%) did not return follow-up questionnaires 3 months post ICU stay, despite reminder telephone calls. We did not find any demographic data differences between responders and non-responders.

Non-responders had higher early psychological scores than responders, indicating worse psychological well-being. Median PTSS-10 score among non-responders was 25 versus 20 among responders (*p* < 0.02), median HADS anxiety subscale score was 5.5 versus 3 (*p* < 0.02) and median HADS depression subscale score was 6 versus 4 (*p* < 0.01).

## Discussion

In this prospective follow-up study we found significant correlations between reported symptoms of post-traumatic stress, anxiety and depression in the week after ICU discharge and 3 months later. The predictive precision of early assessment was best for post-traumatic stress symptoms with an AUROC of 0.90 (Fig. 2).

Using the cut-off values based on our data, relatively few individuals who later suffered from clinically significant psychological symptoms would have remained unidentified as possible cases at the early screening. The negative predictive values were 91% for the HADS depression subscale, 95% for the HADS anxiety subscale and 98% for PTSS-10 part B, indicating that only 2–9% of patients scoring above a threshold of 29 (PTSD), 5 (anxiety) or 4 (depression) at the early assessment would have clinically significant symptoms of post-traumatic stress, anxiety or depression 3 months later. Psychological problems have been shown to be persistent within the first year after ICU stay, indicating long-term disability, with major potential benefit from early detection [6, 18].

Our results imply that psychological screening in the first week after ICU stay could generate high-risk cohorts, including most patients with clinically significant post-traumatic, anxiety or depressive symptoms 3 months post ICU stay. In parallel, many assessed patients could be ruled out as low-risk patients, less likely in need of psychological ICU follow-up. As stated, early post-ICU follow-up has been recommended [12, 19]. However, ICU follow-up resources are limited and supportive measures need to be concentrated on patients in need. With the suggested ward screening cut-off values, 48% of patients in our cohort would have been classified as low-risk patients with no need for further psychological follow-up (data not shown).

Methods for predicting or detecting psychological problems in ICU survivors have been called for [12, 19] and have been addressed in earlier studies [20–24]. The predictive value of PTSS-10 has been assessed before, but only in patients after prolonged mechanical ventilation [25]. Wade et al. [23] developed and validated a new instrument, the intensive care psychological assessment tool (IPAT), to be used in patients with an ICU stay longer than 48 hours and who were awake and capable of answering the questions. The predictive values for later psychological morbidity were moderate, with lower sensitivity and specificity for both outcomes compared with those in this study.

While these and other earlier studies [20–24] indicate that psychological problems can be predicted or detected post ICU stay, patient selection has typically been limited to specific subgroups of ICU survivors. In contrast, our study assesses a wide spectrum of ICU patients with an ICU stay as short as 24 hours and without requiring mechanical ventilation. To our knowledge, this is the first study of early psychological screening of ICU survivors with such a general approach.

Assessing ICU survivors in the ward after ICU stay is becoming more common (personal communication, Stockholm County ICU Follow-up Network) and has been recommended [12, 19]. The HADS and PTSS-10 are fairly short compared with more extensive diagnostic tools and can be administered to patients without engaging a psychologist or psychiatrist. The short, questionnaire-based assessment with these instruments could be supportive in decision-making when considering further psychological follow-up. Such follow-up could be concentrated on patients at high risk for persisting psychological problems.

The early, protocolised psychological evaluation after ICU discharge may also be of value from a research perspective. The potential benefits of ICU follow-up have been difficult to substantiate in clinical trials [26, 27]. Typically, inclusion in such trials has been relatively broad and has not targeted ICU survivor populations with documented high risk for adverse outcome [11, 26]. The screening methods evaluated in our study could be used as a tool to enrich study populations in future intervention studies.

We further hypothesise that an initial first triage, followed by in-depth evaluation and interventions in patients with high early scores, could improve the longer-term psychological outcome in a mixed ICU survivor population. This needs to be investigated in studies combining early screening with early interventions.

In a post-hoc analysis we found that multiple traumatic memories from the ICU (PTSS-10 part A) were associated with a higher degree of post-traumatic stress (PTSS-10 part B) than none or one traumatic memory. This is consistent with earlier findings and strengthens the validity of the PTSS-10 [28].

### Limitations

We did not use interviews to assess diagnostic criteria for post-traumatic stress, anxiety or depression in our study, which is a limitation. Instead, patients returned questionnaires by postal mail.

Another limitation is the use of the PTSS-10 rather than the PTSS-14, a questionnaire with four additional questions developed to adhere to the Diagnostic and Statistical Manual of Mental Disorders-IV (DSM-IV) criteria for PTSD. The reason for using the PTSS-10 was that this was the questionnaire in clinical use at our ICU follow-up clinic at the time of data collection. Both the PTSS-10 and the PTSS-14 have shown good validity in detecting symptoms of PTSD in ICU survivors according to the DSM-IV criteria [13, 22]. The PTSS-10 and HADS questionnaires have been well studied and are considered clinically valuable tools in assessing the degree of post-traumatic stress, anxiety and depressive symptoms, and correlate fairly well with formal diagnostic evaluations. The short questionnaires can be regarded as screening instruments that lead to formal in-depth assessment in patients with high scores, rather than replacing such assessment.

Finally, the response rate was 62% despite reminder letters, a common response rate in ICU follow-up studies [21, 29]. Non-responders had significantly higher early scores for post-traumatic stress, anxiety and depression. Reasons for non-participation are unclear, but considering the high scores in the early assessment we cannot rule out that they may in part have been related to avoidance.

## Conclusions

Screening with the Post-Traumatic Stress Symptoms Checklist-10 and the Hospital Anxiety and Depression Scale in the week after ICU discharge correlates with 3-month outcomes in a wide range of mixed ICU survivors, with fairly good predictive accuracy. The instruments are easily administered and can be of value for identifying ICU survivors at risk for symptoms of post-traumatic stress, anxiety and depression 3 months after ICU discharge. From an ICU follow-up resource allocation perspective, further psychological follow-up can be concentrated on smaller high-risk groups.

## Abbreviations

AUROC: Area under the receiver operating characteristic curve
HADS: Hospital Anxiety and Depression Scale
ICU: Intensive care unit
IQR: Interquartile range
PTSD: Post-traumatic stress disorder
PTSS-10: Post-Traumatic Stress Symptoms Checklist-10
ROC: Receiver operating characteristic
